# Tutorial: dos and don’ts in clinical prediction research for venous thromboembolism

**DOI:** 10.1016/j.rpth.2024.102480

**Published:** 2024-06-18

**Authors:** Banne Nemeth, Mark J.R. Smeets, Suzanne C. Cannegieter, Maarten van Smeden

**Affiliations:** 1 Department of Clinical Epidemiology, Leiden University Medical Center, Leiden, the Netherlands; 2 Department of Thrombosis and Hemostasis, Leiden University Medical Center, Leiden, the Netherlands; 3 Julius Center for Health Sciences and Primary Care, University Medical Center Utrecht, Utrecht University, Utrecht, the Netherlands

**Keywords:** methods, model development, prediction model, risk assessment, validation study, venous thromboembolism

## Abstract

Clinical prediction modeling has become an increasingly popular domain of venous thromboembolism research in recent years. Prediction models can help healthcare providers make decisions regarding starting or withholding therapeutic interventions, or referrals for further diagnostic workup, and can form a basis for risk stratification in clinical trials. The aim of the current guide is to assist in the practical application of complicated methodological requirements for well-performed prediction research by presenting key dos and don’ts while expanding the understanding of predictive research in general for (clinical) researchers who are not specifically trained in the topic; throughout we will use prognostic venous thromboembolism scores as an exemplar.

## Essentials

• Clinical prediction modeling has become a popular domain of venous thromboembolism research.
• This paper presents key dos and don’ts in clinical prediction research.
• It is of great importance to establish the need for a prediction model.
• Realize that a series of steps should be taken before a model is ready to be used.

## 1 Introduction

Clinical prediction modeling has become a popular domain of research in venous thromboembolism (VTE) research. Generally, a distinction is made between diagnostic and prognostic prediction models. The first type of model estimates the probability, for individual patients, of a VTE being present (but undiagnosed or neither completely ruled in or ruled out) at that particular point in time. Examples of these types of models are the Wells and YEARS criteria [1,2]. Prognostic models are instead used to estimate the probability that a patient will develop a VTE within a certain time frame, of which examples are the Padua Prediction and the Caprini score for hospitalized medical patients [3,4]. Besides diagnosing and predicting VTE, prediction models can help healthcare providers make decisions regarding starting or withholding prophylactic/therapeutic interventions, refer for further diagnostic workup, and form a basis for risk stratification in clinical trials.

VTE can be prevented by administration of chemical thromboprophylaxis, which comes at a risk of (major) bleeding [5]. Therefore, exposure to chemical thromboprophylaxis is only warranted in patients in whom the risk of VTE outweighs that of bleeding. In the last decades, earlier prognostic studies have identified several prognostic factors (Table) that can be used to predict VTE risk, such as immobilization, surgery, cancer, and use of oral contraceptives [6]. Also, biomarkers such as D-dimer [7], factor (F)VIII activity [8], and genetic predictors such as FV Leiden mutation [9] are increasingly accessible and add valuable information to an individual’s VTE risk profile. In all, the need for risk estimation in clinical practice and the availability of factors with potentially strong prognostic and diagnostic information make prediction of VTE relevant and feasible and, hence, an appealing approach toward better patient care.

**Table:** Prediction research terminology.

Terms	Meaning
Impact study	Randomized controlled study in which the impact of a prediction model, with subsequent intervention, is trialed.
Implementation	Implementing the prediction model in routine clinical practice.
Model coefficients	Each predictor in a prediction model has a coefficient. This is the value by which the prognostic index (Y) of the model increases for 1 unit increase (or from 0 to 1 for dichotomous predictors) of the predictor.
Optimism	Meaning that the predictive performance measures are too optimistic because of overfitting.
Overfitting	Meaning that the model coefficients are too closely fitted (overfitted) on the derivation sample. This could result in a poor fit of the model in new populations.
Predictive ability of a predictor	The strength of the association between a predictor and the outcome of interest.
Predictive performance of a model	
Discrimination	Performance measure of the prediction model, which indicates its ability to distinguish patients who will develop the outcome of interest from those who will not.
Calibration	Performance measure of the prediction model, which assesses how well predicted probabilities align with observed proportions.
Predictors, prognostic factors	Variables included in the multivariable prediction model.
Updating	Changing the intercept and/or coefficients of the prediction model based on new data. Including new predictors in the model is also a form of updating.

While prediction research has become increasingly popular in recent years, several papers have shown methodological shortcomings and a lack of uniform and adequate reporting for many of these models [10,11]. Moreover, there is a lack of validation studies and, consequently, limited information about the performance of the prediction models in clinical practice [5,12]. For example, at least 13 risk prediction models for VTE for hospitalized medical patients have been published so far, which are hardly used in current clinical practice due to, possibly, among other reasons, lack of good discriminative abilities, lack of proper validation studies, and reluctance of healthcare providers to use such scores (eg, due to personal beliefs) [[13], [14], [15]]. This situation clearly illustrates the strong interest in this topic but also an overgrowth of unused prediction models, whereas the primary goal of most of these models, ie, adequate prevention of VTE in high-risk patients, is still out of reach.

The Transparent Reporting of a multivariable prediction model for Individual Prognosis Or Diagnosis (TRIPOD) statement was designed to improve the reporting of prediction research [16]. In addition, the recently published Prediction model Risk Of Bias ASsessment Tool (PROBAST) was designed to assess the risk of bias and applicability of prediction model studies [17]. The aim of the current guide is to assist in the practical application of complicated methodological requirements for well-performed prediction research. We will do this by presenting key dos and don’ts while expanding the understanding of predictive research in general for (clinical) researchers who are not specifically trained in the topic; throughout, we will use prognostic VTE scores as an exemplar.

## 2 Prediction Life Cycle

**Figure 1:**
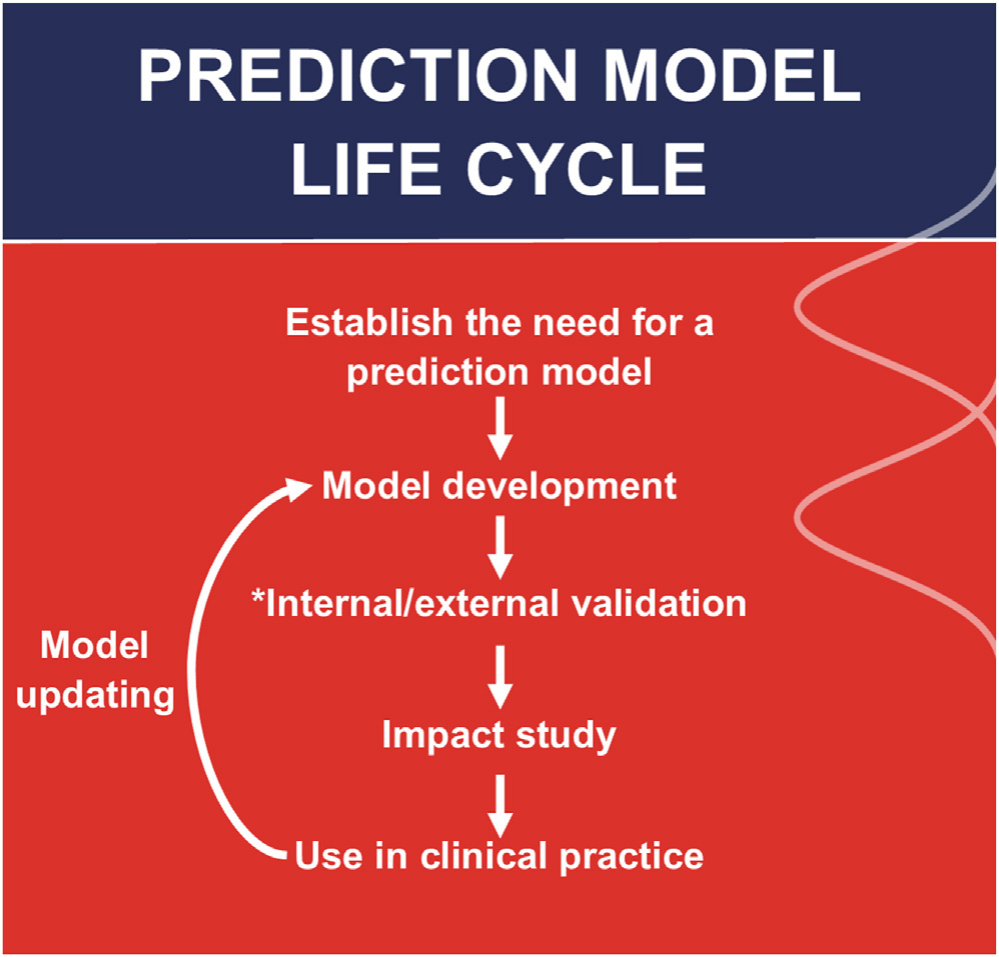
Prediction model life cycle. ∗Both internal and external validation are part of model development. Model updating can take place at any stage after internal/external validation.

To develop a prediction model, a series of steps should be taken before it is ready to be used. This often means that multiple studies have to be performed (Figure 1). Additionally, since the prediction model can be seen as a medical device, implementation (Table) in clinical practice is also dependent on compliance with existing regulations.

## 3 Establish the Need for A Prediction Model

**Table:** 

	
- Establish the need for a prediction model - Define a clear purpose, target population, and time horizon - Find and critically appraise other prediction models that have been developed within the same domain	- Develop a new model when similar models already exist

Just like in other types of research, the initiation of a study (in this case, the development of a prediction model) starts with a hypothesis. For prediction research, this hypothesis is generally that a prediction model can improve clinical decision-making and thereby improve patient outcomes (eg, deciding which hospitalized patients will benefit from thromboprophylaxis and which can go safely without). To get the aim of the prediction model clear, it is necessary to formulate the purpose to predict the target population and to specify the time horizon. Let us assume there is a clinical need to predict the occurrence of VTE (the purpose) in patients hospitalized in an academic center for treatment of a certain medical condition but not receiving thromboprophylaxis (the target population). It is decided that for this patient population, the relevant outcome is VTE occurrence up to 3 months after the first day of hospitalization (the time horizon). For the inclusion of patients, we need to keep in mind the primary aim of the model that is to estimate individual risks for VTE among patients who are not already on thromboprophylaxis because of, for example, a previous VTE.

Before embarking on the task of developing a new prediction model, a literature search should be performed to determine whether a similar prediction model already exists, for instance, by performing a systematic review of the prediction model landscape [18]. Clearly, developing a new prediction model defies the purpose when an existing prediction model with a similar purpose, target population, and time horizon is already available but needs to be further evaluated and possibly refined. Authors should consider first critically appraising and validating previously developed prediction models and, if necessary, consider performing model updating by reestimating/adjusting some or all of the models’ coefficients or adding additional predictors (Table). This way, any newly available data are used most efficiently, as new information is added to the information (based on the development data and any validation studies) that is already embedded in the existing model. A testing procedure for model updating is discussed in detail elsewhere [19].

An example of a situation in which new models were constantly added rather than building on the existing ones is that of models aimed at identifying hospitalized medical patients at high risk for VTE. At least 13 such models exist, of which only a limited number have so far received proper external validation [13]. Consequently, none of these models have been uniformly integrated into guidelines (instead, it is generally advised to assess the risks of VTE [12,20]) and, hence, have not been widely implemented in clinical practice [5,12].

## 4 Data Availability

**Table:** 

	
- Assess data/study type - Calculate the required sample size	- Develop a model if the data/study type does not meet prediction aims - Develop a prediction model if the sample size is unsatisfying

### 4.1 Do the data match the prediction aims?

The design of data collection should match the anticipated prediction aims. We consider here 4 groups of commonly used methods of data collection and their suitability for development of prediction models.

- *Cross-sectional data collection*: can be suitable for the development of diagnostic prediction models to quantify the presence of a certain target disease at the moment of prediction but is almost never suitable for developing prognostic prediction models. For most prognostic prediction studies, it is necessary to know the value of a predictor (Table) before the outcome of interest occurs. In cross-sectional studies, this is not the case, as both predictor and outcome are measured simultaneously. In such a study design, the value of a predictor can even be influenced by the outcome. An example in which cross-sectional data can be used would be a study on the predictive value of genetic variants and the risk of VTE since the genetic variants were surely present before the VTE.
- *Case-control data collection*: absolute risk estimates cannot directly be obtained from a case-control study (unless the data are from a nested case-control study [21]), making it generally less suitable for development of prediction models. Furthermore, when data are obtained through patient questionnaires, researchers need to be aware of recall bias. Nevertheless, an important strength of case-control studies is that the number of cases is generally much higher than in any cohort design; this may allow for a larger number of potential predictors to be considered. Hence, case-control studies can be valuable as so-called predictor finding studies (sometimes called prognostic factor studies [22], Table), which aim to identify individual or a combination of predictors that predict the outcome of interest.
- *Prospective data collection*: prospective cohort studies and randomized controlled trials (RCTs) are often considered preferable study types for prediction research as data are collected similarly to how this would be done following implementation. One drawback of cohort studies might be typically incompleteness of some of the variables in the data set. For example, when we look at development studies of models designed to estimate the risk of VTE recurrence, for the DASH score [23], only 802/1818 (44%) patients had complete data compared with 629/929 (68%) for the Vienna model [24] and 336/646 (52%) for the HERDOO2 model [25]. This led to a substantial loss of data (lower sample size), contributing to an increased risk of overfitting and optimism (Table) [26]. Furthermore, limiting the cohort to complete cases can only lead to selection bias, which can affect model performance [27]. While RCT data are usually more complete than cohort studies, the in- and exclusion criteria of most RCTs can limit their generalizability [28]. In addition, RCTs are prone to large treatment effects, which can greatly hamper model performance [29]. Approaches to account for treatment effects in prediction models have been discussed elsewhere [30,31].
- *Routine healthcare data collection*: large registries such as claims databases and electronic patient record data are increasingly available and used for prediction model development. Although routine healthcare data usually come with a relatively large sample size, registry studies are prone to misclassification and missing data, which also can hamper model development and may affect overall predictive performance (Table) of the model [32].

In general, the population that has been used for model development must (ideally) closely resemble the population at the point of intended application of the model. If these populations differ, it is likely that the predictive performance is compromised due to deviations in the incidence of the predicted outcome or variations in patient characteristics between both populations, known as case-mix differences [33]. A discussion about the use of datasets from multiple populations (eg, multicenter studies) is found elsewhere [34].

### 4.2 Sample size

Simplified rules of thumb (for example, the events per variable ratio) to calculate the minimal sample size for prediction models have been widely implemented in the literature [35]. However, these rules have been shown to be inappropriate for sample size criteria for prediction models, as they are not based on convincing scientific reasoning [36] and perform poorly in large-scale simulation studies [[37], [38], [39]]. Therefore, several new sample size calculation approaches, with accompanying software to simplify the calculations, have recently been developed by leading researchers in the field [[40], [41], [42]]. Because these calculations take the number of candidate predictors, the total sample size, and the events fraction into account, we recommended using these new approaches.

## 5 Preparing the Data

**Table:** 

	
- Perform a literature study on possible candidate predictors - Be aware of predictor measurement heterogeneity - Balance practical aspects and expected predictive value - Consider performing multiple imputation	- Dichotomize predictors without absolute need - Solely include candidate predictors based on univariable association with the outcome - Exclude candidate predictors because of a noncausal relationship with the outcome

### 5.1 Selecting candidate predictors

Predictor variables that are considered for inclusion in the prediction model at the start of development, so-called candidate predictors, can be of various types, such as patient demographics (eg, age and sex), patient history (eg, heart failure and previous VTE), biomarkers (eg, D-dimer), and genetic phenotypes (eg, FV Leiden mutation). Ideally, candidate predictors have a strong expected incremental predictive value (ie, a strong correlation with the outcome on top of other known predictive factors). Information about the predictive ability (Table) of predictors can be obtained from predictor finding studies [22], which comprise a large portion of the prediction modeling literature [11], from earlier published risk prediction models, meta-analyses, and expert opinion. Predictors do not have to be causally related to the outcome of interest to be valuable predictors. A classic example of this is that gray hair is an excellent predictor of mortality, even though there is no causal relationship. Similarly, an example related to VTE would be *socioeconomic status*, which has no obvious direct causal relation to the development of VTE but has been shown to have value in *predicting* VTE [43].

To select candidate predictors, one should balance between (expected) predictive value and practical aspects of measuring the predictor in the setting where the prediction model will be applied. For instance, predictors that are invasive, expensive, or time-consuming to measure may not be adequate for inclusion in a prediction model whose intended point of application is a primary care setting (ie, a thrombin generation assay might function as a good predictor but is impractical compared with a point-of-care D-dimer test). The measurement of the predictor in the study setting should also mimic that in the situation where it is applied as closely as possible to prevent *measurement heterogeneity* that may severely hamper the performance of the prediction model [44]. For instance, in developing the HERDOO2 score, a rule to guide treatment duration for women with unprovoked venous thrombosis, the authors showed that replacement of the VIDAS D-dimer assay (bioMérieux) with other D-dimer assays decreased the predictive ability of the HERDOO2 score [45]. Another example of measurement heterogeneity applies to discrete (categorical) predictors such as heart failure, which can be classified as either type II, III, or IV. One must make sure the same criteria are used during model development, validation, and practice.

Selection of candidate predictors on the basis of statistical criteria, particularly when based on the univariable association with the predicted outcome of interest, should be avoided as much as possible (variable selection is further discussed in section 6, “MODEL DEVELOPMENT” below).

### 5.2 Predictor modeling

Discrete predictor variables, such as patient sex, use of oral contraceptives, or stroke, can be included in the prediction model as categorical dummy variables with a reference category. For instance, sex can be added as the variable “female,” which takes on the value 1 for females and 0 for males (reference). Categories that are rare or categories in which the outcome does not occur can be collapsed with adjacent categories. An example of this would be genetic variants, as there are many that are associated with a (small) increased risk of VTE, and the prevalence of each variant is low [9]. Hence, including every variant as separate predictor results in a model with many predictors and potential overfitting as result. In this case, when the associations between the variants and VTE are relatively similar, they can be combined in a single predictor.

Categorization of continuous predictor variables (such as patient age or D-dimer value) is generally not advised to avoid unnecessary reduction in the predictive performance of the prediction model, which can amount to the equivalent of discarding one-third of the dataset [46]. Instead, continuous predictors can be added as such in the prediction model, and possible nonlinear relationships can be modeled via fractional polynomials and cubic splines (presentation of more nonlinear and more complex prediction models is discussed below) [47,48]. The predictive ability of a continuous or categorical predictor may vary with the values of other predictors, which can be modeled by adding interaction terms to the prediction model. However, significant interaction terms do not necessarily improve predictive performance, and considering many interaction terms can cause the model to become overfitted [49].

### 5.3 Missing data

Candidate predictors that have missing values can hamper development of a prediction model. With a complete case analysis, only subjects that have complete information on each of the included predictors and outcomes are included. A couple of predictors with a large number of missing values (or a large number of predictors with a small number of missing values) may contribute to a large decrease in the effective sample size in a complete case analysis. For predictors with a large number of missing values, including them as candidate predictors may, therefore, not be worthwhile [50]. Another drawback of a complete case analysis is that it assumes the missing values are missing completely at random, which they seldom are, and this will lead to selection bias in that case [51]. Relying on a less stringent but still critical assumption of data missing at random [52,53], multiple imputation has become an increasingly popular approach to handle missing data on predictors, as it preserves the size of the original data set.

## 6 Model Development

**Table:** 

	
- Choose a suitable statistical model - Be conservative in data-driven selection of predictors - Apply shrinkage - Focus on optimism corrected performance	- Perform univariable, forward, or stepwise predictor selection - Use the Hosmer–Lemeshow test to evaluate calibration performance

### 6.1 Model building

Once all candidate predictors have been selected and missing data have been accounted for, a prediction model can be developed. Both logistic regression and survival models are used for the prediction of binary outcomes. To decide which model should be used, researchers have to determine whether there is significant loss to follow-up (right censoring) in the data [49]. Often, when the follow-up is short, loss to follow-up is negligible, and logistic regression is a valid modeling approach. For example, in a previously published prediction model for VTE in the postpartum period, the authors limited follow-up to 6 weeks following delivery. In this case, a logistic regression was performed [54]. However, when there is significant loss to follow-up, survival analysis is required [49]. In the absence of competing risks (ie, when VTE is the outcome of interest, death is a competing risk), the Cox regression model can be used [55]. When competing risk(s) is present, the Fine–Gray model is recommended [55,56]. An example of such an approach can be found in a study by Pabinger et al. [57], in which the authors developed and validated a prediction model for cancer-associated VTE with significant competing risk. Alternative modeling approaches, such as increasingly popular machine learning techniques (eg, random forest, neural networks, and support vector machines), have yet to show their benefit in the context of clinical prediction models [58].

The prediction model can now be derived by applying the statistical model (or machine learning technique) to the data of all candidate predictor variables included. Before the model is finalized, variable selection is often done to reduce the number of variables in the final prediction model. While variable selection generally does not result in predictive performance benefits, models with fewer predictors can be more user-friendly and practical to use in a clinical setting. However, many popular variable selection strategies, such as univariable selection, forward selection, stepwise, and backward elimination, have been shown to increase the risk of model overfitting [[59], [60], [61]]. To reduce this risk, the general advice is to use conservative approaches to variable selection, such as backward elimination with a high *P* value criterion (eg, a *P* value of .20) [49]. Additionally, one may force some (well-established) candidate predictors in the final model regardless of their predictive performance.

By applying regression shrinkage approaches (often called “regularization” in the context of machine learning), the risk of model overfitting, which occurs when the prediction model captures idiosyncrasies in the data that do not generalize to other settings, can be further reduced. In brief, shrinkage approaches introduce a small bias in the regression coefficients, generally toward the zero effect, to reduce prediction error. Several approaches to shrinkage, such as uniform shrinkage, Ridge, Lasso, and Firth’s correction, have been suggested [47,62,63]. For more details about shrinkage, we refer to Pavlou et al. [64].

### 6.2 Model performance measures

For prediction models with a binary outcome, the performance of the prediction model is often expressed in terms of risk calibration, discrimination (Table), and overall performance [65]. Performance measures for prediction models with more than 2 categories are described elsewhere [66].

**Figure 2:**
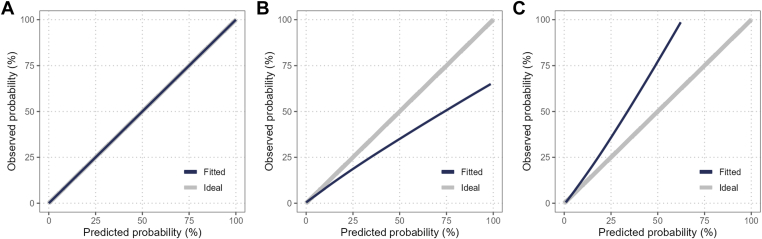
(A) Model with perfect calibration, (B) model that overestimates the risks, and (C) model that underestimates the risks.

Calibration is the ability of the prediction model to accurately estimate the risks (do *x* of 100 subjects with a predicted risk of x% truly experience the predicted event?). A common way to investigate calibration is by plotting a calibration curve that depicts the relation between estimated risks (horizontal axis) vs the observed outcome frequencies (vertical axis). In Figure 2, we show 3 different calibration plots. Figure 2A depicts a model with perfect calibration. In Figure 2B, C, a model is shown that systematically over- and underestimates the risk of patients compared with the observed risks, respectively [67]. Often, summary measures of the calibration, ie, the calibration intercept and slope, are also reported [65]. The closer the calibration intercept is to 0, the better, as this means that the predicted average risk is similar to the average risk observed in the population. For the calibration slope, the optimal value is 1. However, this value can be misleading as a model can have a slope of 1, even when it systematically over- or underestimates the risks [67]. Hence, the calibration plot should always be shown. We advise against the use of the Hosmer–Lemeshow statistic for checking the calibration as it is known to have many drawbacks, including low statistical power [68], making it an unreliable approach.

Discrimination is the ability of the prediction model to discriminate between individuals who develop an event vs those who do not. For logistic regression models (binary outcomes), a common statistic for discrimination is the concordance probability or C statistic (area under the receiver operating characteristic curve), which is determined by comparing the predicted risk of each patient in the population who developed the outcome with those who did not [69]. This C statistic then represents the proportion of instances in which the predicted risk is higher for patients who do develop the outcome. So, a C statistic of 0.5 means that the model is just as likely to assign a higher risk to patients who do and do not develop the outcome. Thus, the closer the C statistic is to 1, the better the discrimination. In the setting of time-to-event outcomes, the concordance probability is often called the C index [47]. This C index is determined in a similar way as the C statistic, with the addition that it is now also taken into account whether patients who developed the outcome earlier have a higher predicted risk than patients who developed the outcomes later in time. Again, the closer a C index is to 1, the better the performance of the model. Generally, reporting the C statistic or C index with the 95% CI is sufficient, and no additional information is conveyed by also reporting the receiver operating characteristic curve.

Other measures of overall performance, such as the Brier score and pseudo-R-squared, are also commonly used to quantify model performance. Measures that quantify the incremental value of predictors or compare models (eg, the Net Reclassification Index [70]) and measures that quantify clinical usefulness (eg, decision curve analyses [71]) are beyond the scope of this text.

## 7 Model Validation

**Table:** 

	
- Perform an internal validation (or internal-external) and external validation before implementation	- Perform an internal validation by random split sample - Validate the model on datasets with fewer than 100 events

When developing a prediction model, internal validation is essential to obtain valid estimates of predictive performance. This is because estimates of performance on the same data that were used to develop the prediction model tend to be too optimistic [49]. With bootstrap (repeated sampling with replacement of individuals from the original dataset) or cross-validation procedures (repeated partitioning of the dataset into a larger set to develop the model and a smaller set to evaluate it), where all the prediction modeling steps (including the selection of variables) are repeated several times, the optimism of the predictive performance measures can be investigated and “corrected” to obtain more realistic measures of performance [49].

Model validation can also be done by splitting the data into meaningful groups. For instance, if the prediction model is derived from data obtained from multiple academic centers, the natural unit for splitting is by center. This is called internal-external validation [72]. Every center is left out once for validation of the prediction model that is based on the remaining studies. In contrast, split-sample approaches that randomly split the data into training (to fit the model) and test sets (to validate the model) should be avoided because they are statistically inefficient and a weak test of model performance [73,74].

It is widely acknowledged that, next to any internal validations, a prediction model needs to be assessed in external validation studies with independent data. For external validation studies, a dataset that contains at least 100 subjects who experienced the event has been recommended as a minimum sample size [75]. More precise estimates of the required sample size can be obtained with the method(s) described by Riley et al. [76]. For any external validation study, the degree of relatedness between the setting where the model was developed and where it was validated should be carefully reported to facilitate the interpretation of findings [33]. Furthermore, it should be noted that a prediction model can never be conclusively “validated” and that, preferably, before implementation in a specific population, the model should be validated locally [77]. Lastly, following validation, the effectiveness and safety of implementing the clinical prediction model (CPM) in practice needs to be established. In such an impact study (Table) design, the combination of the CPM performance (ie, the predicted risks as provided by the CPM) and the subsequent treatment options (which depend on the predicted risks) will be investigated [78,79].

## 8 Reporting

**Table:** 

	
- Follow the TRIPOD statement for model reporting	- Forget to report the full model parameters, including the intercept

The TRIPOD statement provides guidance on the key items to report when describing, developing, evaluating, validating, or updating (Table) clinical prediction models [16]. Numerous systematic reviews of prediction models have shown that adequate reporting is often lacking to an extent that external validation based on the reported information is often not possible [10,11]. Following the TRIPOD reporting guideline when developing a prediction model for VTE is therefore recommended.

Reporting of the prediction model should clearly summarize all modeling steps taken to derive the model and the characteristics of the final model, including the full model equation with intercept and regression coefficients. Reporting should also provide sufficient detail on the moment and time horizon of prediction and collection of data, such as the design of data collection (see above), as well as the methods and timing of predictor and outcome measurements. Estimates of predictive performance corrected for optimism should also be reported for readers to understand the potential predictive power of the prediction model.

Further, to facilitate the implementation of prediction models, Bonnett et al. [80] have summarized 4 methods on how prediction models can be presented using a point score system, graphical score chart, nomogram, or application/website. For a detailed explanation of the advantages and disadvantages (including clinical examples), we refer to their article [80].

## 9 Concluding Remark

In this article, we describe some of the key dos and don’ts when developing a prediction model. Prediction model development is only the starting point of the life cycle of a prediction model. It requires external validation studies, implementation, and impact studies [79] before it is ready to be implemented in clinical practice.
